# Implementation of a Low-Cost Navigation System Using Data Fusion of a Micro-Electro-Mechanical System Inertial Sensor and an Ultra Short Baseline on a Microcontroller

**DOI:** 10.3390/s25103125

**Published:** 2025-05-15

**Authors:** Julian Winkler, Sabah Badri-Hoeher

**Affiliations:** Department of Computer Science and Electrical Engineering, Kiel University of Applied Sciences, Grenzstraße 5, 24149 Kiel, Germany; sabah.badri-hoeher@fh-kiel.de

**Keywords:** MEMS inertial sensor, ultra short baseline, autonomous underwater vehicle, square-root Kalman filter

## Abstract

In this work, a low-cost low-power navigation solution for autonomous underwater vehicles is introduced utilizing a Micro-Electro-Mechanical System (MEMS) inertial sensor and an ultra short baseline (USBL) system. The complete signal processing is implemented on a cheap 16-bit fixed-point arithmetic microcontroller. For data fusion and calibration, an error state Kalman filter in square root form is used, which preserves stability in case of rounding errors. To further reduce the influence of rounding errors, a stochastic rounding scheme is applied. The USBL measurements are integrated using tightly coupled data fusion by deriving the observation functions separately for range, elevation, and azimuth angles. The effectiveness of the fixed point implementation with stochastic rounding is demonstrated on a simulation, and the the complete setup is tested in a field test. The results of the field test show an improved accuracy of the tightly coupled data fusion in comparison with loosely coupled data fusion. It is also shown that the applied rounding schemes can bring the fixed-point estimates to a near floating point accuracy.

## 1. Introduction

Autonomous Underwater Vehicles (AUVs) have revolutionized oceanographic research and exploration by enabling autonomous operations in deep, inaccessible, and hazardous underwater environments. A pivotal challenge in their deployment, however, lies in achieving precise underwater navigation. Unlike terrestrial or aerial systems, AUVs cannot reliably utilize Global Navigation Satellite Systems (GNSSs) due to rapid signal attenuation in water, restricting GNSS availability to brief surface intervals [1]. Consequently, navigation predominantly relies on inertial systems such as Fiber-Optic Gyroscopes (FOGs) in combination with dead reckoning based on Doppler Velocity Logs (DVLs). While these systems offer high accuracy, their substantial cost—particularly for high-precision FOG and DVL units—poses a significant barrier to affordability, especially for smaller AUVs, where they may constitute over 50% of total platform expenses.

To bridge this gap, recent advancements focus on integrating low-cost Micro-Electro-Mechanical System (MEMS)-based inertial sensors with acoustic positioning through sensor fusion techniques, such as Kalman filtering [2].

In this work, we present a novel, cost-effective navigation solution for AUVs combining MEMS-based inertial measurement with acoustic positioning, optimized for real-time operation on low-power microcontrollers. By prioritizing computational efficiency without sacrificing performance, this system aims to democratize AUV access for scientific and commercial applications—from marine biodiversity mapping to underwater infrastructure inspection—where high-cost solutions remain prohibitive. The integration of modular, low-power hardware further ensures scalability across AUV classes, underscoring our goal to redefine the balance between precision, affordability, and operational versatility in underwater navigation.

## 2. Related Work

Underwater navigation makes extensive use of inertial navigation. Because pure inertial navigation is not applicable for more than a few minutes [3], it is normally integrated in multi-sensor data fusion [4].

Combined navigation and sensor data fusion in underwater applications is mostly done using extended Kalman filters [5,6,7]. In [8], an unscented Kalman filter is used to model the distribution of quantization noise. For three-dimensional navigation, error state Kalman filters are used in combination with quaternion calculations, as detailed in [9]. Such filters are typically implemented with 32-bit or 64-bit floating-point calculation. Before sufficient calculation power was available, Kalman filters were often implemented in square root form [10,11]. Square root Kalman filters are typically used for simple systems with a small number of optimizable states. With digital signal processors capable of performing millions of fixed-point multiplications per second, it has become viable to use square root Kalman filters for complex navigation and data fusion tasks, while embedded class processors are often much more cost and power efficient than application class processors.

In [12], a navigation solution for AUVs is proposed based on tightly coupled data fusion that combines an inertial sensor and DVL. A chi-square detection-aided dual-factor adaptive filter is used to suppress outliers. Ref. [13] proposes Inertial Navigation System (INS)/DVL data fusion using a square-root unscented information filter to ensure the symmetry and positive definiteness of the covariance matrix or information matrix and to enhance the stability of the filter. Ref. [14] proposes a virtual beam predictor based on multi-output least-squares support vector regression in order to allow for data fusion when not all DVL beams are available. In [15], an extended exponential weighted Kalman filter is combined with a long short-term memory neural network to make the DVL/INS data fusion more robust against outliers. These advanced filtering algorithms often come with high computational complexity and are therefore difficult to realize in a small, low-cost AUVs. Therefore, ref. [16] proposed a low-cost AUV navigation solution using an extended Kalman filter of only five dimensions in combination with complementary filtering.

In summary, this literature review highlights the importance of having high calculation precision. Our proposed method combines low-cost hardware with advanced implementation methods.

While data fusion between MEMS inertial sensors and USBL is well-established [16,17,18], our contribution focuses on implementing this fusion using 16-bit fixed-point arithmetic and analyzing the impact of rounding errors in this context. Our method is able to effectively reduce the influence of rounding errors introduced by 16-bit fixed point representation. The reduced calculation cost allows us to use a high-dimensional state vector without losing real-time capabilities when implemented on an inexpensive microcontroller. This can, for example, be used to perform calibration and data fusion with the same filter.

## 3. System Overview

The navigation solution used in this work consists of a Seatrac X150 Ultra-Short Baseline (USBL) beacon, an Advanced Navigation Spatial MEMS inertial sensor, and a Microchip dsPIC33CH512MP508 microcontroller. For laboratory tests, the MEMS sensor was replaced by a much cheaper Bosch BNO055. The microcontroller and the MEMS sensor were directly connected via an RS232 interface, and acceleration, gyroscope, and magnetic measurements were sent to the microcontroller with a sampling rate of 100 Hz. The microcontroller’s main loop also operated at 100 Hz, synchronizing itself to the MEMS sampling rate. A second RS232 interface linked the microcontroller to the AUV’s main PC to transmit the position and velocity estimates. This connection also facilitated the transfer of additional data, such as USBL pings, to the microcontroller and enabled the storage of raw MEMS measurements for post-processing. When using the BNO055 sensor, an I2C interface was employed to connect it to the microcontroller. The dsPIC33CH microcontroller family is a dual-core system, with both cores featuring DSP units. In our setup, one core was dedicated to real-time signal processing, while the other handled communication with the host PC and the MEMS sensor.

## 4. Data Fusion Method

A common way to describe the position and motion of a vehicle in three-dimensional space is an indirect or error state Kalman filter combining a quaternion equation and linear algebra equations, as described in detail in [9]. The proposed data fusion method uses an error state Kalman filter with a 15-dimensional state vector x including position p, velocity v, rotation vector θ, accelerometer bias $a_{b}$, and gyroscope bias $\omega_{b}$. Each of them is three-dimensional.

(1) $$x={\left[\begin{array}{ccccc} p & v & \theta & a_{b} & \omega_{b} \end{array}\right]}^{⊤}$$

The measurement input vector $u_{m}$ consists of the accelerometer measurement $a_{m}$ and the gyroscope measurement $\omega_{m}$.

(2) $$u_{m}={\left[\begin{array}{cc} a_{m} & \omega_{m} \end{array}\right]}^{⊤}$$

For the nominal state, the rotation vector θ is replaced by unit quaternion q{θ}, where q{·} is the quaternion exponential map. Quaternion multiplications are written as $q=q_{1}⊗q_{2}$ in order to be distinguished from normal matrix multiplications.

The state transition $x_{k+1}=f\left(x_{k}\right)$ is defined in the Equations (3)–(5), where the index *k* marks the current time step and k+1 marks the next time step of a time discrete system. Δt is the sampling period, g is the gravity vector, and R{θ} is the exponential map of the rotation vector θ as rotation matrix.

(3) $$\begin{array}{cc} p_{k+1} & =p_{k}+v_{k}\Delta t+\frac{1}{2}\left(R\left\{\theta_{k}\right\}\left(a_{m,k}-a_{b,k}\right)+g\right){\Delta t}^{2} \end{array}$$

(4) $$\begin{array}{cc} v_{k+1} & =v_{k}+\left(R\left\{\theta_{k}\right\}\cdot \left(a_{m,k}-a_{b,k}\right)+g\right)\Delta t \end{array}$$

(5) $$\begin{array}{cc} q\{\theta_{k+1}\} & =q\left\{\theta_{k}\right\}⊗q\left\{\left(\omega_{m,k}-\omega_{b,k}\right)\cdot \Delta t\right\} \end{array}$$

The state transition function can be linearized around a given working point of x and $u_{m}$ into a matrix $F_{x}$ and $F_{u}$ such that $x_{k+1}=F_{x,k}\cdot x_{k}+F_{u,k}\cdot u_{m,k}$. Similar to [9], $F_{x}$ and $F_{u}$ can be derived as shown in Equations (6) and (7). I is the 3×3 identity matrix and ${\left[\cdot \right]}_{\times }$ is the skew operator, such that $a\times b={\left[a\right]}_{\times }\cdot b$. The rotation matrix is written as R=R{θ}.

(6) $$\begin{array}{l} F_{x}={\left.\frac{\partial f}{\partial \delta x}\right|}_{x,u_{m}}=\\ \left[\begin{array}{ccccc} I & I\Delta t & 0 & 0 & 0\\ 0 & I & -{\left[R\left(a_{m}-a_{b}\right)\right]}_{\times }\Delta t & -R\Delta t & 0\\ 0 & 0 & I & 0 & -R\Delta t\\ 0 & 0 & 0 & I & 0\\ 0 & 0 & 0 & 0 & I \end{array}\right] \end{array}$$

(7) $$\begin{array}{cc} \; & F_{u}={\left.\frac{\partial f}{\partial \delta u_{m}}\right|}_{x,u_{m}}=\left[\begin{array}{cc} 0 & 0\\ R\Delta t & 0\\ 0 & R\Delta t\\ 0 & 0\\ 0 & 0 \end{array}\right] \end{array}$$

For data fusion and filtering, the state vector x will be estimated on each time step *k* as a state estimate vector ${\hat{x}}_{k}$ with a covariance matrix $P_{k}$. The Kalman filter splits the estimation into a prediction and correction step. The prediction step is defined as shown in Equations (8) and (9), where Q is the covariance matrix of u. The prediction step gives the a priori estimate marked with index k|k−1.

(8) $$\begin{array}{cc} {\hat{x}}_{k|k-1} & =F_{x}\cdot {\hat{x}}_{k-1}+F_{u}\cdot u_{k} \end{array}$$

(9) $$\begin{array}{cc} P_{k|k-1} & =F_{x}\cdot P_{k-1}\cdot F_{x}^{⊤}+F_{u}\cdot Q\cdot F_{u}^{⊤} \end{array}$$

Given an external observation of the system state y=h(x) and the associated observation matrix $H={\left.\frac{\partial h}{\partial \delta x}\right|}_{x}$ and observation covariance matrix V, the correction step can be defined as shown in Equations (10)–(12). The intermediate matrix K is the Kalman gain.

(10) $$\begin{array}{cc} K_{k} & =P_{k|k-1}H^{⊤}{\left(HP_{k|k-1}H^{⊤}+V\right)}^{-1} \end{array}$$

(11) $$\begin{array}{cc} {\hat{x}}_{k} & ={\hat{x}}_{k|k-1}+K_{k}\left(y-H\cdot {\hat{x}}_{k|k-1}\right) \end{array}$$

(12) $$\begin{array}{cc} P_{k} & =\left(I-K_{k}H\right)P_{k|k-1} \end{array}$$

The Kalman filter steps can be transferred into a square root form. In this work, the square root form of Cholesky factors is used. The Cholesky factor of a matrix M is here written as $M^{1/2}$, such that $M=M^{1/2}\cdot M^{⊤/2}$. To create the square root form, all covariance matrices are replaced by their Cholesky factors. As shown in [19], the prediction step in square root form can be written as shown in Equation (13) where $S=P^{1/2}$ is the Cholesky factor of the covariance of the state estimate and G is an orthogonal matrix used to compress S back in its original size.

(13) $$\left[\begin{array}{c} {\left(S_{k|k-1}\right)}^{⊤}\\ 0 \end{array}\right]=G\left[\begin{array}{c} {\left(F_{x}\cdot S_{k-1}\right)}^{⊤}\\ {\left(F_{u}\cdot Q^{1/2}\right)}^{⊤} \end{array}\right]$$

The correction step is transferred into its square root form according to [19], resulting in Equation (14).

(14) $$\begin{array}{cc} \; & \left[\begin{array}{ccc} {\left(V+HP_{k}H^{⊤}\right)}^{⊤/2} & \; & {\left(V+HP_{k}H^{⊤}\right)}^{⊤/2}K_{k}^{⊤}\\ 0 & \; & S_{k}^{⊤} \end{array}\right]\\ \; & =G\left[\begin{array}{ccc} V^{⊤/2} & \; & 0\\ S_{k|k-1}^{⊤}H^{⊤} & \; & S_{k|k-1}^{⊤} \end{array}\right] \end{array}$$

After extracting K and V from the result of Equation (14), the new state estimate $\hat{x}$ can be obtained the same way as in Equation (11).

Each time when calculating Equations (13) and (14), an orthogonal transformation represented by G has to be found, which creates the required zeros in the bottom left part of the result matrix. This is the same process as applying a QR-decomposition, which splits a square matrix into a triangular and an orthogonal part. The orthogonal matrix can be discarded, and the triangular matrix corresponds to the left hand side of Equation (13) or (14). There are multiple methods for performing the QR-decomposition. The most common methods utilize either Givens rotations or Householder transformations. The method using Householder transformations is in general more efficient for non-sparse matrices, as it can be applied per column instead of per element. Therefore, Householder transformations are used in this work.

### 4.1. QR Decomposition Using the Householder Transformation

**Figure 1:**
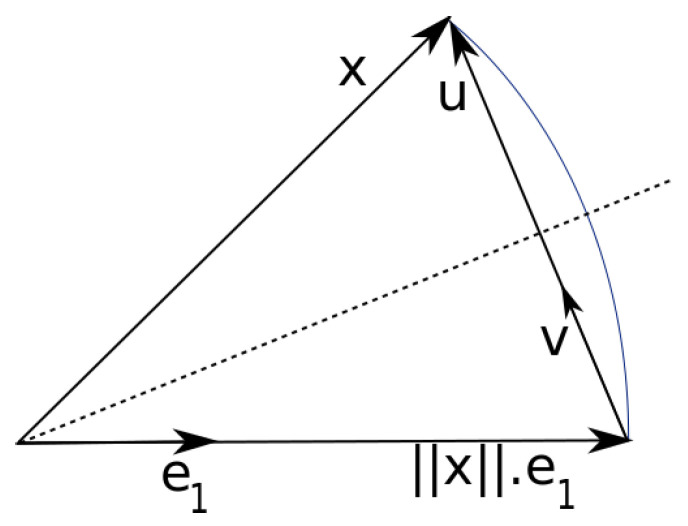
Householder reflection for QR decomposition [21].

As described in [20], the Householder transformation can be performed as a series of Householder reflections, where each column vector of a matrix M is mirrored in the same hyperplane. As shown in Figure 1, the column vector is called x, the normal vector of the hyperplane is u, and the basis vector of the target direction is $e_{1}$. To create a triangular matrix, the reflection plane must be positioned in such a way that all elements of the first column vector except the first are 0 and the column vector lies on the first basis vector $e_{1}$. Because the amounts of the column vectors must not change during an orthogonal transformation, the first column vector x must therefore become $\pm \left||x|\right|\cdot e_{1}$ after the transformation.

The normal vector u of the hyperplane can be found by subtracting the desired first column vector $\pm \left||x|\right|\cdot e_{1}$ from the current first column vector x, as shown in Equation (15). The normalized normal vector v is then calculated from this, as shown in Equation (16).

(15) $$\begin{array}{cc} u & =x\pm \left||x|\right|\cdot e_{1} \end{array}$$

(16) $$\begin{array}{cc} v & =\frac{u}{\left||u|\right|} \end{array}$$

The new sign for the first element of the new column vector is arbitrary, but it should be the opposite of the previous sign. This prevents the vector u from becoming very small, leading to rounding errors.

$$u=x+\mathrm{sgn}\left(x_{1}\right)\cdot \left||x|\right|\cdot e_{1}$$

To mirror each column vector x on the plane with the normal vector v, x must first be projected onto v and then subtracted twice from x.

$$x\leftarrow x-2v\left(v^{⊤}x\right)$$

In each step, two square root operations are required to determine the amounts of x and v. The second root operation can be saved by using u directly instead of v.

$$x\leftarrow x-2\frac{u}{\left||u|\right|}\left({\left(\frac{u}{\left||u|\right|}\right)}^{⊤}x\right)=x-2u\frac{u^{⊤}x}{{\left||u|\right|}^{2}}=x-2u\frac{u^{⊤}x}{u^{⊤}u}$$

The same process can then be applied to matrix M again, except that one column and one row are omitted each time. This is repeated until M becomes an upper triangular matrix.

$$\left[\begin{array}{cccc} x & x & x & x\\ x & x & x & x\\ x & x & x & x\\ x & x & x & x \end{array}\right]\overset{Q_{1}}{\to }\left[\begin{array}{cccc} x & x & x & x\\ 0 & x & x & x\\ 0 & x & x & x\\ 0 & x & x & x \end{array}\right]\overset{Q_{2}}{\to }\left[\begin{array}{cccc} x & x & x & x\\ 0 & x & x & x\\ 0 & 0 & x & x\\ 0 & 0 & x & x \end{array}\right]\overset{Q_{3}}{\to }\left[\begin{array}{cccc} x & x & x & x\\ 0 & x & x & x\\ 0 & 0 & x & x\\ 0 & 0 & 0 & x \end{array}\right]$$

### 4.2. Fixed-Point Implementation

To implement the whole filter on a microcontroller with only fixed point arithmetic, a new data structure has been designed to hold the Cholesky factor S of the covariance of state estimate, which adds an additional per-row exponent to the matrix containing only fixed point representations of its elements. As shown in Equation (17), the matrix S is split into a diagonal exponent matrix E containing exponents with base 2 and a mantissa matrix M containing fixed point values. This way, the E matrix can be ignored during the QR-decomposition, because it is diagonal and thus also triangular. Only the M matrix needs to be QR-decomposed. This means that the computation-intensive Householder transforms can be calculated completely in fixed point arithmetic, while the complete S matrix still has some advantages of floating point numbers.

(17) $$\begin{array}{cc} S & =E\cdot M=\left[\begin{array}{ccc} 2^{\;e_{1}} & 0 & 0\\ 0 & 2^{\;e_{2}} & 0\\ 0 & 0 & 2^{\;e_{3}} \end{array}\right]\cdot \left[\begin{array}{ccc} m_{1,1} & m_{1,2} & m_{1,3}\\ m_{2,1} & m_{2,2} & m_{2,3}\\ m_{3,1} & m_{3,2} & m_{3,3} \end{array}\right]\\ \; & =\left[\begin{array}{ccc} m_{1,1}\cdot 2^{\;e_{1}} & m_{1,2}\cdot 2^{\;e_{1}} & m_{1,3}\cdot 2^{\;e_{1}}\\ m_{2,1}\cdot 2^{\;e_{2}} & m_{2,2}\cdot 2^{\;e_{2}} & m_{2,3}\cdot 2^{\;e_{2}}\\ m_{3,1}\cdot 2^{\;e_{3}} & m_{3,2}\cdot 2^{\;e_{3}} & m_{3,3}\cdot 2^{\;e_{3}} \end{array}\right] \end{array}$$

### 4.3. Stochastic Rounding

When using limited precision values like 16-bit signed integers for the mantissas in M, each multiplication of two of these numbers results in a 32-bit integer, which needs to be rounded to be stored in a 16-bit register again. With the typical Round to Nearest (RN) approach, this can lead to some unfavorable effects, as the rounding errors often sum up quickly over the iterations of the filter. By replacing the RN method with a Stochastic Rounding (SR) method, the behavior can be made much more predictable, as the accumulated rounding error over time can now be described with a random walk function [22], whose expected magnitude over time follows a square root function. In addition to multiplications, the Householder algorithm also requires square root and division operations whose results have to be rounded as well. The SR implementations for the different cases are shown in Algorithms 1–3. The given pseudo code uses ≫ to represent bit-wise right shift and ≪ for bit-wise left shift. PRNG32() and PRNG16() are 32-bit and 16-bit Pseudorandom Number Generator (PRNG) functions.
**Algorithm 1** Rounding last n bits of 32-bit integer with stochastic rounding**function** ROUND16_STOCHASTIC(*x*, *n*)    mask←(1≪n)−1    p←PRNG32()&mask    residual←x&mask    x←x≫n    **if** p<residual **then**        x←x+1    **end if**    **return** *x***end function**

**Algorithm 2** Integer division with stochastic rounding
**function** DIVISION_STOCHASTIC($x_{1}$, $x_{2}$)    $x_{1}\leftarrow x_{1}+\left(\left(x_{2}\cdot \mathrm{PRNG}16\left(\right)\right)\gg 16\right)$    **return** $x_{1}/x_{2}$
**end function**



**Algorithm 3** Integer square root with stochastic rounding
**function** SQRT_STOCHASTIC(value)    result←0    exp←0x8000    **while** exp≠0 **do**        temp←result|exp        **if** (temp·temp)≤value **then**           result←temp        **end if**        exp←exp≫1    **end while**                                                                ▹ rounding last bit with stochastic rounding    ${\mathrm{temp}}_{32}\leftarrow \left(\mathrm{result}\ll 16\right)+\mathrm{PRNG}16\left(\right)$    **if** $(\left({\mathrm{temp}}_{32}\cdot {\mathrm{temp}}_{32}\right)\gg 32)<\mathrm{value}$ **then**        result←result+1    **end if**    **return** result
**end function**



To illustrate the effectiveness of the stochastic rounding method, a simplified abstract system is constructed. The system consists of a three-dimensional state vector and uses an identity matrix as the state transition model. At each iteration, the filter receives a one-dimensional observation—a linear combination of the system states—with a constant standard deviation. Following each update, the predicted state uncertainty is evaluated using the filter’s covariance matrix, or the square root of the covariance matrix in the case of the fixed-point implementation. The deviation is expected to go down as the filter incorporates more observations over time.

**Figure 2:**
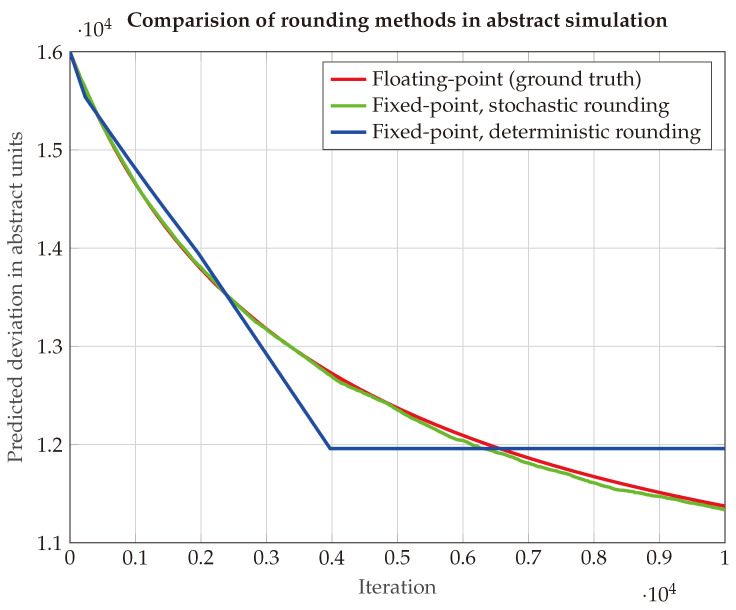
Abstract simulation of covariance matrix evolution on simplified three-state Kalman filter. Comparison between floating-point implementation as ground truth (red), fixed-point stochastic rounding (green), and fixed-point deterministic rounding (blue).

As can be seen in Figure 2, the SR implementation result is much closer to the result from floating-point arithmetic. Because of the low precision of the 16-bit numerical representation, there is only a very limited amount of possible step sizes. It can be clearly seen that the RN implementation utilizes the nearest possible step size, but it diverges quickly from the floating-point results.

**Figure 3:**
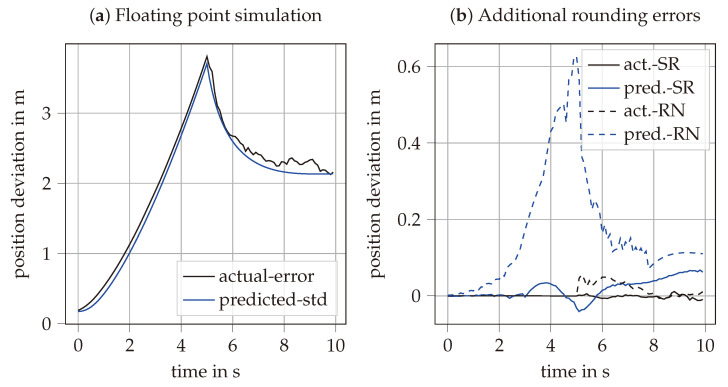
Simulation results with (**a**) floating point arithmetic and (**b**) fixed point arithmetic. The first 5 s reflect pure inertial drift, followed by a phase with an additional position measurement. The predicted standard deviation is shown in blue, and the actual deviation in black, with the RN rounding method represented by dashed lines. All results are averaged over 100 simulations.

To assess the impact of rounding errors in a more realistic scenario, the following setup was simulated. The initial three-dimensional position, velocity, and orientation are assumed to be known. Then, for 5 s, only noisy inertial measurements are available. After the first 5 s, an additional noisy position measurement becomes available. The three-dimensional angular velocity measurement has a noise spectral density of $3\times 10^{-7}\;\mathrm{rad}/s/\sqrt{\mathrm{Hz}}$, and the acceleration measurement has a noise spectral density of $0.3\;m/s^{2}/\sqrt{\mathrm{Hz}}$. The additional noisy position measurement has a noise spectral density of $1.5\;m/\sqrt{\mathrm{Hz}}$. Figure 3a shows the simulation results for floating-point arithmetic, averaged over 100 repeated simulations, with the absolute actual error plotted against the predicted error. In the first 5 s, the position estimation uncertainty increases mainly because of the acceleration measurement noise. After the position measurement becomes available, the uncertainty stabilizes at above 2 m error. Figure 3b shows the difference between the results with floating point and fixed point arithmetic. Consistent with Figure 2, the SR method provides significantly more accurate predicted uncertainty compared to RN. This difference has minimal impact on the actual error during the pure inertial phase. However, when the position measurement is available, the inaccurately predicted uncertainty affects data fusion, resulting in a slight increase in the actual error.

### 4.4. Tightly Coupled Data Fusion with USBL

The data fusion with USBL measurements can be implemented either using a Loosely Coupled (LC) or Tightly Coupled (TC) approach [23]. The TC method is chosen here because it allows us to have separate certainty values for the measurement of range and angle. The known beacon position in global coordinates $p_{\mathrm{beacon}}$ is first transferred to the body coordinate system, as shown in Equation (18). Then, the observation vector $y={\left[\begin{array}{ccc} r_{m} & \beta_{az,m} & \beta_{el,m} \end{array}\right]}^{⊤}$ including range, azimuth angle, and elevation angle can be predicted using the h(x) function given in Equation (19).

(18) $$\begin{array}{cc} p_{B} & =R^{⊤}\left\{\theta \right\}\left(p_{\mathrm{beacon}}-p\right) \end{array}$$

(19) $$\begin{array}{cc} h & =\left[\begin{array}{c} \left|\right|p_{B}\left|\right|\\ \mathrm{atan}2\left(p_{B,y},\;p_{B,x}\right)\\ \mathrm{atan}2\left(p_{B,z},\;||p_{B,xy}\left|\right|\right) \end{array}\right] \end{array}$$

This function is then linearized around the current state estimate to obtain the observation matrix $H_{x}$ given in Equation (20).

(20) $$H_{x}={\left.\frac{\partial h(x)}{\partial x}\right|}_{x}=\left[\begin{array}{ccccc} \frac{\partial h}{\partial p} & 0 & \frac{\partial h}{\partial \theta } & 0 & \ldots \end{array}\right]$$

Here, the derivative of the observation vector relative to the position vector is given by $\frac{\partial h}{\partial p}=-\frac{\partial h}{\partial p_{B}}R^{⊤}\left\{\theta \right\}$, with the derivative of the observation relative to the beacon position in body coordinates given in Equation (21). The derivative of the observation relative to the rotation vector θ is given by $\frac{\partial h}{\partial \theta }=\frac{\partial h}{\partial p_{B}}R^{⊤}\left\{\theta \right\}{\left[p_{\mathrm{beacon}}-p\right]}_{\times }$.

(21) $$\begin{array}{cc} \frac{\partial r}{\partial p_{B}} & =\frac{p_{B}}{\left|\right|p_{B}\left|\right|}\\ \frac{\partial \beta_{az}}{\partial p_{B}} & =\frac{\left[\begin{array}{ccc} p_{B,y} & -p_{B,x} & 0 \end{array}\right]}{\left|\right|p_{B,xy}{\left|\right|}^{2}}\\ \frac{\partial \beta_{el}}{\partial p_{B}} & =\frac{\left[\begin{array}{ccc} -\frac{p_{B,z}\cdot p_{B,x}}{\left|\right|p_{B,xy}\left|\right|} & -\frac{p_{B,z}\cdot p_{B,y}}{\left|\right|p_{B,xy}\left|\right|} & \left|\right|p_{B,xy}\left|\right| \end{array}\right]}{\left|\right|p_{B}{\left|\right|}^{2}}\\ \frac{\partial h}{\partial p_{B}} & =\left[\begin{array}{c} \frac{\partial r}{\partial p_{B}}\\ \frac{\partial \beta_{az}}{\partial p_{B}}\\ \frac{\partial \beta_{el}}{\partial p_{B}} \end{array}\right] \end{array}$$

## 5. Sensor Calibration

While the sensor calibration process typically requires precise reference measurement systems, the previously described Kalman filter can also be extended to estimate the sensors’ orientation together with calibration parameters. This way, the calibration process can be performed without additional reference measurements. Therefore, the state estimate x of Equation (1) has to be extended as shown in Equation (22), where $a_{s}$ and $\omega_{s}$ are the sensitivity correction factors for each axis of the accelerometer and the gyroscope, and $\alpha ={\left[\begin{array}{ccc} \alpha_{yz} & \alpha_{zy} & \alpha_{zx} \end{array}\right]}^{⊤}$ and $\gamma ={\left[\begin{array}{cccccc} \gamma_{yz} & \gamma_{zy} & \gamma_{zx} & \gamma_{xy} & \gamma_{xy} & \gamma_{yx} \end{array}\right]}^{⊤}$ are the axis misalignment angles for the accelerometer and gyroscope.

(22) $$x={\left[\begin{array}{ccccccccc} p & v & \theta & a_{b} & \omega_{b} & a_{s} & \omega_{s} & \alpha & \gamma \end{array}\right]}^{⊤}$$

To define the axis misalignment angles, a reference coordinate system is needed. This reference system is defined as the Accelerometer Orthogonal Frame (AOF) [24], where the measured accelerometer x-axis lies directly on the x-axis of the AOF. The y-axis of the AOF is fixed to be in the plane spanned by the x-axis and y-axis of the accelerometer measurement axes. The z-axis of the AOF is then given solely by the orthogonality of the system. Therefore, three calibration angles are needed to align the accelerometer to the AOF, and six calibration angles are needed to align the gyroscope. An accelerometer measurement vector $a_{m}$ can be transformed into the AOF by multiplying it matrix $T_{a}$ as shown in Equation (23). Similarly, the gyroscope measurement is aligned as shown in Equation (24).

(23) $$\begin{array}{cc} a_{\mathrm{AOF}} & =T_{a}\cdot a_{m}=\left[\begin{array}{ccc} 1 & -\alpha_{yz} & \alpha_{zy}\\ 0 & 1 & -\alpha_{zx}\\ 0 & 0 & 1 \end{array}\right]\cdot a_{m} \end{array}$$

(24) $$\begin{array}{cc} \omega_{\mathrm{AOF}} & =T_{g}\cdot \omega_{m}=\left[\begin{array}{ccc} 1 & -\gamma_{yz} & \gamma_{zy}\\ \gamma_{xy} & 1 & -\gamma_{zx}\\ -\gamma_{xy} & \gamma_{yx} & 1 \end{array}\right]\cdot \omega_{m} \end{array}$$

The state transition f(x) previously defined in Equations (3)–(5) is extended as shown in Equations (25)–(27), where the ⊙ operator represents an elementwise multiplication between two vectors.

(25) $$\begin{array}{cc} p_{k+1} & =p_{k}+v_{k}\Delta t+\frac{1}{2}\left(R\left\{\theta_{k}\right\}T_{a}a_{k}+g\right){\Delta t}^{2} \end{array}$$

(26) $$\begin{array}{cc} v_{k+1} & =v_{k}+\left(R\left\{\theta_{k}\right\}T_{a}a_{k}+g\right)\Delta t \end{array}$$

(27) $$\begin{array}{cc} q\{\theta_{k+1}\} & =q\left\{\theta_{k}\right\}⊗q\left\{T_{g}\omega_{k}\Delta t\right\}\\ a_{k} & =a_{s}⊙a_{m,k}-a_{b}\\ \omega_{k} & =\omega_{s}⊙\omega_{m,k}-\omega_{b} \end{array}$$

The new state transition is linearized around a given working point of x, as shown in Equation (28), replacing the $F_{x}$ previously defined in (6).

(28) $$F_{x}={\left.\frac{\partial f}{\partial \delta x}\right|}_{x,u_{m}}=\left[\begin{array}{ccccccccc} I & I\Delta t & 0 & 0 & 0 & 0 & 0 & 0 & 0\\ 0 & I & F_{1} & F_{2} & 0 & F_{4} & 0 & F_{6} & 0\\ 0 & 0 & I & 0 & F_{3} & 0 & F_{5} & 0 & F_{7}\\ 0 & 0 & 0 & I & 0 & 0 & 0 & 0 & 0\\ 0 & 0 & 0 & 0 & I & 0 & 0 & 0 & 0\\ 0 & 0 & 0 & 0 & 0 & I & 0 & 0 & 0\\ 0 & 0 & 0 & 0 & 0 & 0 & I & 0 & 0\\ 0 & 0 & 0 & 0 & 0 & 0 & 0 & I & 0\\ 0 & 0 & 0 & 0 & 0 & 0 & 0 & 0 & I \end{array}\right]$$

where submatrices $F_{1}$ to $F_{7}$ are defined as follows:

$$\begin{array}{cc} F_{1} & =-{\left[R\left\{\theta \right\}T_{a}a\right]}_{\times }\Delta t\\ F_{2} & =-R\left\{\theta \right\}T_{a}\Delta t\\ F_{3} & =-R\left\{\theta \right\}T_{g}\Delta t\\ F_{4} & =-R\left\{\theta \right\}T_{a}\cdot \mathrm{Diag}\left(a_{m}\right)\Delta t\\ F_{5} & =-R\left\{\theta \right\}T_{g}\cdot \mathrm{Diag}\left(\omega_{m}\right)\Delta t\\ F_{6} & =R\left\{\theta \right\}\left[\begin{array}{ccc} -a_{y} & a_{z} & 0\\ 0 & 0 & -a_{z}\\ 0 & 0 & 0 \end{array}\right]\Delta t\\ F_{7} & =R\left\{\theta \right\}\left[\begin{array}{cccccc} -\omega_{y} & \omega_{z} & 0 & 0 & 0 & 0\\ 0 & 0 & -\omega_{z} & \omega_{x} & 0 & 0\\ 0 & 0 & 0 & 0 & -\omega_{x} & \omega_{y} \end{array}\right]\Delta t \end{array}$$

Often, integrated MEMS sensors come with integrated magnetometers. The magnetometer can be included in the calibration, as shown in [25]. Since the magnetometer measurements are not involved in the prediction step, the state transition function and transition matrix are not effected.

## 6. Results

**Figure 4:**
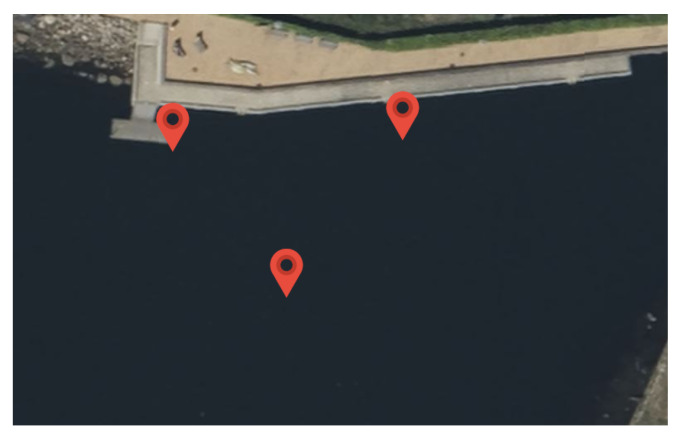
Field test with an AUV at the Kiel Fjord.

**Figure 5:**
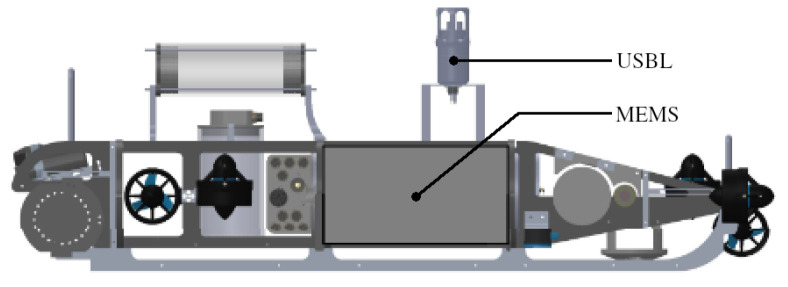
Positioning of navigational sensor on the AUV used in the field test.

For the field test, the AUV was driving close to the surface on a triangular path marked with buoys, as shown in Figure 4. The test side was located in the Kiel Fjord, and the triangle had a side length of around 30 m. The water depth at the test side was between 3 m and 8 m. The vehicle used for the test was a custom-designed AUV with a length of about 1 m and a weight of about 50 kg. The placement of the navigational sensors is shown in Figure 5. It is noted that both sensors were relative close to each other in the horizontal direction, so rotations around the vertical axes had less impact on the lever arm. The vehicle was driving in non-autonomous mode using a tethering cable, and the actual path was recorded from above the water to measure the reference position.

**Figure 6:**
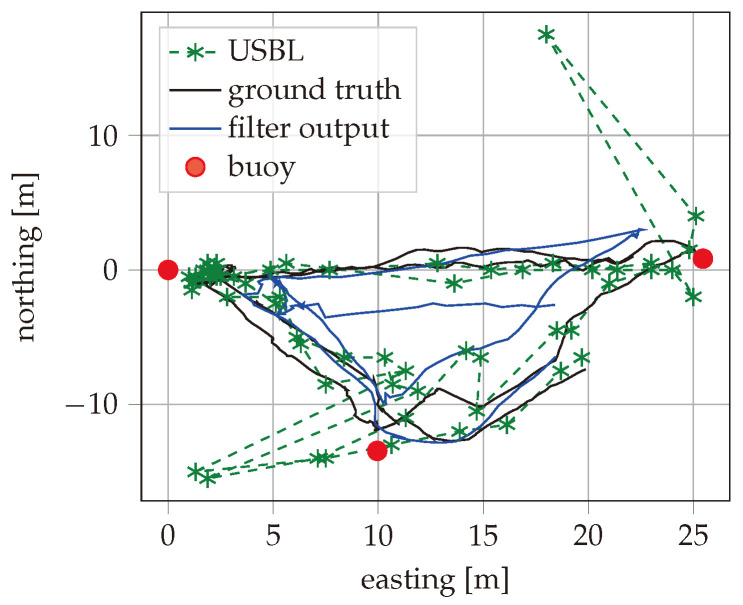
Estimated position during the field test plotted northing over easting in comparison with the ground truth and USBL samples.

**Figure 7:**
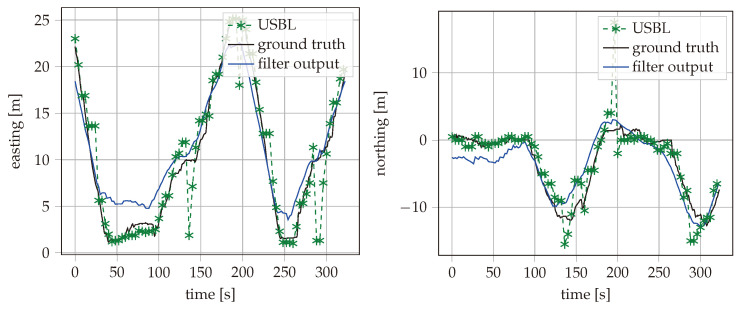
Estimated position during the field test plotted over time in comparison with the ground truth and USBL samples.

During the field test, the AUV was driving for about an hour while recording data from MEMS and USBL. As our method performs joined calibration and data fusion, the accuracy grows over time. For this reason, only the position accuracy during a 10 min time period at the end of the test was taken into account for the evaluation. The recorded reference position, the USBL fixes, and the estimated vehicle path with best filter configuration are shown in Figure 6 for the time period used for evaluation. The east and north positions are also plotted separately over time in Figure 7. Different configurations of the described filter are applied to the data recorded from the field test. The maximum error and the Root Mean Squared Error (RMSE) are calculated for the estimated positions over the same 10 min time period. The filter has been processed with different types of input data enabled. This includes inertial measurements of the MEMS, the USBL measurements with either TC or LC data fusion, and feedback of the thruster set points. Additionally, the implementation method can be floating-point or fixed-point with SR or RN rounding methods. The results are summarized in Table 1. It can be seen that the RMSE of the filter output is close to the RMSE of the raw USBL measurements, despite the USBL measurements being only available in a 5 s interval. In addition, the maximum error is strongly reduced from 17.62 m to 6.09 m. It can be seen that the SR method clearly reduces the precision difference between the 16-bit fixed-point and 32-bit floating-point implementations. The RMSE of the SR fixed-point implementation is only 5% higher than with the floating-point results. In contrast, the RN implementation had a 16% higher error. The comparison with a few-state Kalman filter extended with complementary filtering, as described in [16], showed a clear advantage of our proposed method. The complementary filtering setup was limited to LC USBL data fusion. As shown in Table 1, the maximum error was almost identical between the complementary filtering setup and our method with LC USBL data fusion, while our method with TC USBL data fusion had a reduced maximum error.

An advantage of the proposed method is its ability to perform the calibration jointly with the data fusion. To test the effectiveness of this approach, the estimated bias parameters of the MEMS sensor were compared between a standalone calibration in the laboratory and a joined calibration during the field test. The results are presented in Table 2. It can be seen that the estimated gyroscope bias parameters closely match between the two calibrations. The accelerometer bias parameters differ strongly, which is also reflected in the estimation uncertainty of around $5\times 10^{-2}\;\frac{m}{s^{2}}$. The accelerometer calibration works much better in the laboratory test because the sensor can be freely rotated. In the field test, the sensor always has the z-axis aligned with the gravity of the earth. For the same reason, the sensitivity and orthogonality parameters are difficult to estimate in a short field test. Therefore, these parameters were fixed during the field test and not included in the comparison.

The existing methods can be broadly classified into three categories. The first relies on high-precision sensors, offering high accuracy at a high cost [2,4,12]. The second uses low-cost MEMS sensors combined with complex, high-rate, many-state data fusion algorithms, which reduce hardware costs but impose significant computational load [6]. The third category also employs low-cost sensors, but it minimizes computational demand through simplified data fusion techniques [16]. Our method belongs to the second category, with the key advantage of offloading the data fusion to a dedicated signal processing unit. This approach eliminates the computational burden on the application processor, making its full processing power available for other tasks. A comparison of the advantages and disadvantages can be seen in Table 3.

## 7. Conclusions

This work presents a novel implementation method to achieve near-floating-point precision on a limited 16-bit microcontroller, enabling the development of a low-cost, low-power standalone navigation system. While the microcontroller’s power consumption may be minor in the overall power management of an AUV, this approach offers the advantage of enhanced numerical stability, regardless of computational constraints. The implementation leverages a square-root form of the error state Kalman filter to establish a numerically stable inertial navigation system that allows for data fusion from external sensors. The implementation has been adapted and optimized for fixed-point calculation to be used on a 16-bit microcontroller. A stochastic rounding method has been applied to increase the precision of the fixed-point implementation. The algorithm has been adapted for TC USBL data fusion. Additional optimizable states have been added for calibration of bias, sensitivity, and orthogonality errors of the MEMS sensor.

The proposed implementation has been tested on different calibration and data fusion tasks. Data collected from a field test have been used to compare the performance of different filter configurations. It was shown that the accuracy of the fixed-point calculation could be brought close to the floating-point performance using the SR method. Also, the use of TC data fusion with USBL and the inclusion of thruster feedback led significant error reduction.

## Figures and Tables

**Table 1:** Results with different data fusion configurations.

Type	MEMS	USBL	Thrust	RMSE	Max. Error
		raw		3.51 m	17.61 m
float	yes	TC	yes	3.18 m	6.09 m
float	yes	TC		4.5 m	13.38 m
float	yes	LC	yes	5.28 m	8.67 m
float	yes	LC		7.02 m	17.07 m
fix16 SR	yes	TC	yes	3.36 m	6.21 m
fix16 RN	yes	TC	yes	3.69 m	6.75 m
complementary filter [16]	yes	LC	yes	3.62 m	8.54 m

**Table 2:** Comparison between standalone calibration in the laboratory and joined calibration during the field test.

Element	Standalone Calibration	Joined Calibration
accelerometer bias x	$-8.73\times 10^{-2}$ $\frac{m}{s^{2}}$	$2.95\times 10^{-2}\;\frac{m}{s^{2}}\pm \;6.58\times 10^{-2}\;\frac{m}{s^{2}}$
accelerometer bias y	$-3.51\times 10^{-2}$ $\frac{m}{s^{2}}$	$6.39\times 10^{-1}\;\frac{m}{s^{2}}\pm 4.83\times 10^{-2}\;\frac{m}{s^{2}}$
accelerometer bias z	$-8.77\times 10^{-3}\;\frac{m}{s^{2}}$	$-9.64\times 10^{-2}\;\frac{m}{s^{2}}\pm 4.41\times 10^{-2}\;\frac{m}{s^{2}}$
gyroscope bias x	$2.50\times 10^{-3}\;\frac{\mathrm{rad}}{s}$	$2.53\times 10^{-3}\;\frac{\mathrm{rad}}{s}\pm 8.20\times 10^{-6}\;\frac{\mathrm{rad}}{s}$
gyroscope bias y	$-4.31\times 10^{-4}\;\frac{\mathrm{rad}}{s}$	$-4.82\times 10^{-4}\;\frac{\mathrm{rad}}{s}\pm 8.14\times 10^{-6}\;\frac{\mathrm{rad}}{s}$
gyroscope bias y	$-1.11\times 10^{-3}\;\frac{\mathrm{rad}}{s}$	$-1.21\times 10^{-3}\;\frac{\mathrm{rad}}{s}\pm 1.48\times 10^{-5}\;\frac{\mathrm{rad}}{s}$

**Table 3:** Advantages and disadvantages of different categories of underwater navigation solutions.

Method	Advantages	Disadvantages
Precise sensors	High accuracy	High cost
MEMS sensors + high rate, many states data fusion	Low cost	Limited accuracy High computational load
MEMS sensors + few states, simplified data fusion	Low cost Low computational load	Limited accuracy Suboptimal data fusion
Our method: MEMS sensors + high rate, many-state data fusion on a dedicated signal processing unit	Low cost No computational load on application processor	Limited accuracy

## Data Availability

Data are contained within the article.

## Abbreviations

The following abbreviations are used in this manuscript:

AUV	Autonomous Underwater Vehicle
INS	Inertial Navigation System
FOG	Fiber-Optic Gyroscope
DVL	Doppler Velocity Log
USBL	Ultra-Short Baseline
MEMS	Micro-Electro-Mechanical Systems
RN	Round to Nearest
SR	Stochastic Rounding
PRNG	Pseudorandom Number Generator
AOF	Accelerometer Orthogonal Frame
RMSE	Root Mean Squared Error
TC	Tightly Coupled
LC	Loosely Coupled
GNSS	Global Navigation Satellite System

## References

[1] Alexandris C., Papageorgas P., Piromalis D. (2024). Positioning Systems for Unmanned Underwater Vehicles: A Comprehensive Review. Appl. Sci..

[2] Zhang T., Shi H., Chen L., Li Y., Tong J. (2016). AUV positioning method based on tightly coupled SINS/LBL for underwater acoustic multipath propagation. Sensors.

[3] Oliver J.W. (2007). An Introduction to Inertial Navigation.

[4] Panish R., Taylor M. Achieving high navigation accuracy using inertial navigation systems in autonomous underwater vehicles. Proceedings of the OCEANS.

[5] Loebis D., Sutton R., Chudley J. (2002). Review of multisensor data fusion techniques and their application to autonomous underwater vehicle navigation. J. Mar. Eng. Technol..

[6] Karmozdi A., Hashemi M., Salarieh H., Alasty A. (2020). Implementation of translational motion dynamics for INS data fusion in DVL outage in underwater navigation. IEEE Sens. J..

[7] Nicosevici T., Garcia R., Carreras M., Villanueva M. (2004). A review of sensor fusion techniques for underwater vehicle navigation. MTS/IEEE Techno-Ocean.

[8] Xu J., Li J., Xu S. (2011). Analysis of quantization noise and state estimation with quantized measurements. J. Control Theory Appl..

[9] Solà J. (2017). Quaternion kinematics for the error-state Kalman filter. arXiv.

[10] Park P., Kailath T. (1995). New square-root algorithms for Kalman filtering. IEEE Trans. Autom. Control.

[11] Van Der Merwe R., Wan E.A. The square-root unscented Kalman filter for state and parameter-estimation. Proceedings of the International Conference on Acoustics, Speech, and Signal Processing.

[12] Liu S., Zhang T., Zhang J., Zhu Y. (2021). A new coupled method of SINS/DVL integrated navigation based on improved dual adaptive factors. IEEE Trans. Instrum. Meas..

[13] Guo Y., Wu M., Tang K., Zhang L. (2018). Square-root unscented information filter and its application in SINS/DVL integrated navigation. Sensors.

[14] Jin K., Chai H., Su C., Xiang M. (2023). A performance-enhanced DVL/SINS integrated navigation system based on data-driven approach. Meas. Sci. Technol..

[15] Zhang S., Zhang T., Zhang L. (2023). An underwater SINS/DVL integrated system outlier interference suppression method based on LSTM-EEWKF. IEEE Sens. J..

[16] Liu J., Yu T., Wu C., Zhou C., Lu D., Zeng Q. (2024). A low-cost and high-precision underwater integrated navigation system. J. Mar. Sci. Eng..

[17] Rao J., Chen J., Ding W., Gong Z. Navigation information fusion for an AUV in rivers. Proceedings of the 2012 IEEE International Conference on Multisensor Fusion and Integration for Intelligent Systems (MFI).

[18] Qin H., Wang X., Wang G., Hu M., Bian Y., Qin X., Ding R. (2023). A novel INS/USBL/DVL integrated navigation scheme against complex underwater environment. Ocean Eng..

[19] Simon D. (2006). Optimal State Estimation: Kalman, H∞, and Nonlinear Approaches.

[20] Anderson B., Moore J. (1979). Optimal Filtering.

[21] Commons W. (2011). Householder Reflection for QR Decomposition. https://commons.wikimedia.org/wiki/File%3aHouseholder.svg.

[22] Mikaitis M. Stochastic Rounding: Algorithms and Hardware Accelerator. Proceedings of the 2021 International Joint Conference on Neural Networks (IJCNN).

[23] Jalal F., Nasir F. Underwater Navigation, Localization and Path Planning for Autonomous Vehicles: A Review. Proceedings of the 2021 International Bhurban Conference on Applied Sciences and Technologies (IBCAST).

[24] Tedaldi D., Pretto A., Menegatti E. A robust and easy to implement method for IMU calibration without external equipments. Proceedings of the 2014 IEEE International Conference on Robotics and Automation (ICRA).

[25] Guo P., Qiu H., Yang Y., Ren Z. The soft iron and hard iron calibration method using extended Kalman filter for attitude and heading reference system. Proceedings of the 2008 IEEE/ION Position, Location and Navigation Symposium.

